# The Art of Decorating Wards

**Published:** 1913-01-04

**Authors:** 


### THE ART OF DECORATING WARDS.

### Two Designs at the Sunderland Royal Infirmary.

For some weeks before Christmas the nurses and patients at the Royal Infirmary, Sunderland, were busily employed in preparing the decorations for the wards and corridors, with the result that some of the effects achieved were really beautiful. For instance, the girls' ward was got up to represent "Fairyland," while in the boys' ward a complete Indian encampment was erected. One of our men's medical wards is deserving of special mention, the decorations taking the form of a ship's deck, complete in every detail, even to models of the captain and officers on the bridge, whilst lifebelts, boats, &c., hanging round the ward added to the general effect. The whole of this work was carried out by the patients themselves, and reflected very good taste and ingenuity on their part. For the evergreens the committee are indebted to the Earl of Strathmore, who, year by year, is good enough to send sufficient to enable the staff to decorate on a very lavish scale.

Early on Christmas morning the wards were visited by the nurses, who sang familiar carols, and wished the patients a Happy Christmas. Dinner was served at twelve o'clock, and the resident staff and others were kept busy carving and waiting upon the patients, who seemed thoroughly to enjoy the substantial fare. After dinner the men smokers were supplied with tobacco, the gift of the Mayor of Sunderland, Alderman Stansfield Richardson, J.P., who is also Chairman of the Infirmary General Committee, and Mr. John Mitchinson. The resident medical staff and nurses subsequently had their Christmas dinner in the Lecture Hall. During dinner the institution was visited by the Mayor wearing his chain of office. On Boxing Day there were 196 patients in the wards, and each one was granted permission by the Matron to invite a friend to tea, which was served at 4.30, and, thanks to a number of kind friends, there was an ample supply of good things. After tea, the resident medical and nursing staffs arranged a series of games, and Nurse Olsen, dressed in Danish national costume, sang several songs. The resident medical staff contributed largely to the evening's entertainment—dressed as "A Broken-down Toff," "Charley's Aunt," "A Coal-hewer," and a "Red Indian" they visited the various wards and sang songs and duets. On Saturday, Dec. 28, the Mayor, the ex-Mayor, and a large number of the committee and their friends assembled to witness the distribution from the Christmas trees. Two large trees, given by the Earl of Durham, had been erected in the boys' and girls' wards. At 6 P.M. the trees were lighted, and after several carols had been sung by the nurses, "Santa Claus," senior resident medical officer, made a triumphant entry in his chariot, which was drawn by his colleagues on the staff, all in fancy costume, and, after the merriment which had been caused by his entrance had subsided, he and his colleagues proceeded with the distribution of the gifts from the trees, each patient in the institution receiving a toy and a useful article of clothing. The clothing and toys were all gifts from friends of the institution.

Afterwards an extensive programme, comprising songs, duets, and a musical monologue, kindly arranged by Mr. Newton Laycock, musical director to the Sunderland Education Committee, took place. During the entertainment the Mayor spoke of the admirable manner in which everything had been carried out, thanks to the skill of the Matron, Miss Amour.

